# Evaporation of serum after long-term biobank storage: A chemical analysis of maternal serum from a large Danish pregnancy screening registry

**DOI:** 10.1371/journal.pone.0293527

**Published:** 2023-10-26

**Authors:** Cecilie S. Uldbjerg, Karina M. Sørensen, Christian H. Lindh, Panu Rantakokko, Russ Hauser, Anders Juul, Anna-Maria Andersson, Elvira V. Bräuner

**Affiliations:** 1 Department of Growth and Reproduction, Copenhagen University Hospital—Rigshospitalet, Copenhagen, Denmark; 2 International Centre for Research and Research Training in Endocrine Disruption of Male Reproduction and Child Health (EDMaRC), Rigshospitalet, University of Copenhagen, Denmark; 3 Danish National Biobank, Statens Serum Institut, Denmark; 4 Division of Occupational and Environmental Medicine, Lund University, Lund, Sweden; 5 Department of Health Security, Finnish Institute for Health and Welfare, Kuopio, Finland; 6 Department of Environmental Health, Harvard T.H. Chan School of Public Health, Harvard University, Boston, Massachusetts, United States of America; 7 Department of Clinical Medicine, University of Copenhagen, Copenhagen, Denmark; University of New South Wales, AUSTRALIA

## Abstract

**Background:**

Relying on freezer stored biospecimens is preferred in epidemiolocal studies exploring environmental pregnancy exposures and later offspring health. Storage duration may increase the pre-analytical variability, potentially adding measurement uncertainty. We investigated evaporation of maternal serum after long-term biobank storage using ions (sodium, Na^+^; chloride, Cl^-^) recognized for stability and relatively narrow normal biological reference ranges in human serum.

**Methods:**

A chemical analysis study of 275 biobanked second trimester maternal serum from a large Danish pregnancy screening registry. Serum samples were collected between 1985–1995 and stored at -20°C. Ion concentrations were quantified with indirect potentiometry using a Roche Cobas 6000 analyzer and compared according to storage time and normal biological ranges in second trimester. Ion concentrations were also compared with normal biological variation assessed by baseline Na^+^ and Cl^-^ serum concentrations from a separate cohort of 24,199 non-pregnant women measured *before* freezing with the same instrument.

**Results:**

The overall mean ion concentrations in biobanked serum were 147.5 mmol/L for Na^+^ and 109.7 for Cl^-^. No marked linear storage effects were observed according to storage time. Ion concentrations were consistently high across sampling years, especially for specific sampling years, and a relatively large proportion were outside respective normal ranges in second trimester: 38.9% for Na^+^ and 43.6% for Cl^-^. Some variation in concentrations was also evident in baseline serum used as quality controls.

**Conclusions:**

Elevated ion concentrations suggest evaporation, but independent of storage duration in the present study (27–37 years). Any evaporation may have occurred prior to freezer storage or during the first 27 years. Other pre-analytical factors such as low serum volume have likely influenced the concentrations, particularly given the high *within year* variability. Overall, we consider the biobanked serum samples internally comparable to enable their use in epidemiological studies.

## Introduction

Human biospecimens used in research present opportunities for the purpose of studying environmental exposures and health. Many epidemiological studies make efforts to establish effects of chemical exposures in fetal life on long-term health, which require access to exposure data collected already during the pregnancy. Importantly, several pregnancy cohorts have emerged during past decades, providing unique resources for studying these prenatal exposures [1,2]. Millions of biological samples have been collected as part of these cohorts and are stored in biorepository freezers for future research [3–9]. A growing number of studies have now attempted to accurately measure a range of chemical exposures in biobanked maternal biospecimen in order to provide insights into the role of the prenatal environment on offspring health [10–14]. The usefulness of biobanked samples for epidemiological research many years after its collection largely depends on the extent to which sample preservation has affected the chemical levels [15]. Supposed evaporation from the tubes during years of storage may result in increased concentrations of the analytes of interest, which would then appear erroneously high; a particular concern if storage effects are not similar across samples. Along with storage time, other pre-analytical variations and errors may also introduce differential effects on the quality of biological samples and influence analysis results, including inappropriate sample handling, insufficient mixing of samples, variability in sample container material, geometry, and volume, hemolysis etc. Unfortunately, the pre-analytical phase is rarely taken into consideration in research projects intended to use biobanked samples for estimating chemical exposures.

The Danish Pregnancy Screening Registry is a large cohort with available blood samples from almost 130,000 pregnant women collected through a pregnancy screening program from 1979 until 1996. From 1985 onward, maternal serum was stored at the Danish National Biobank for future research, including a recently initiated project investigating prenatal exposures to endocrine disrupting chemicals and later testicular cancer. In a separate cohort, we have previously observed that serum was affected differently by evaporation over years of storage [16], and we hypothesize that chemical concentrations in this present collection of samples may therefore be skewed toward higher concentrations in older samples, potentially affecting the quality of our samples and validity of future regression analyses.

In this study, we aimed to investigate the effect of long-term storage at -20°C on evaporation by quantifying serum ion concentrations of sodium, Na^+^ and chloride, Cl^-^. Analyses of these closely regulated ions may reveal evaporation dependent changes in concentrations of analytes of interest during long-term storage.

## Methods and materials

### Study design

A chemical analysis study of evaporation of biobanked serum after storage at -20°C by investigating serum concentrations of selected ions (sodium, Na^+^ and chloride, Cl^-^), known to be stable in stored human serum and with much studied and relatively narrow human biological normal ranges.

### Data source and study material

We accessed biobanked maternal serum samples from the Danish Pregnancy Screening Registry, a large cohort of Danish pregnant women who participated in a pregnancy screening program from 1985 until 1996. Blood samples were collected from pregnant women during their second trimester of pregnancy (gestational weeks 14 to 22). The entire collection of samples is maintained at the Danish National Biobank (Statens Serum Institut in Copenhagen, Denmark).

For the present study, a total of 275 serum samples were randomly selected with an even distribution of 25 samples per sampling year (25 consecutive samples of separate individuals from the same randomly selected box of samples from a given year between 1985 to 1995).

### Sample handling and storage conditions

Blood collection for serum was performed once for each pregnant woman. After blood was drawn, samples were allowed to clot. Clotted blood samples were centrifuged at 4°C at 3,000 rpm before the supernatants of serum were pipetted for storage. The sample handling procedure at the time was transport at ambient temperature until freezing. The collection of serum samples is stored at -20°C in 2 ml cryotubes with screw caps. The collection of serum samples contains between 1–2 ml per sample, but some individual tubes may contain less than 1 ml.

### Ion analyses

The concentrations of Na^+^ and Cl^-^ were measured by an indirect potentiometry assay using an ion-selective electrode (Cobas 6000 Roche/Hitachi, Basel, Switzerland), known to provide stable analysis for a wide range of analytes [17]. The analyses were performed in 2022 at the Danish National Biobank, Statens Serum Institute, Copenhagen, Denmark, all in a random single batch. Prior to ion analyses, the samples were thawed at room temperature for ≤2 hours. The samples were hereafter thoroughly mixed on a whirl mixer for at least 5 seconds and then immediately after manually pipetted into Cobas cups (300 μl). The pipette was pre-wetted by aspirating and dispensing an amount of the sample a few times before transferring to the cups. The process of mixing and pipetting was done for batches of 25 samples, such that 25 samples were pipetted followed by ion analysis. The next batch of 25 samples were prepared, while the prior 25 samples were ion analyzed. The samples were automatically diluted by the analytical instrument prior to analysis. All samples were analyzed using the same calibrator. The instrument measured the serum aliquots from the center of the cup and provided an error message in case of insufficient serum volume.

The normal biological ranges in maternal serum during second trimester of pregnancy for each ion are Na^+^_second trimester_: 129–148 mmol/L and Cl^-^_second trimester_: 97–109 mmol/L) [18]. The concentrations of both Na^+^ and Cl^-^ have wider normal ranges during pregnancy compared to non-pregnant adult populations [18].

### Non-frozen (baseline) quality control samples

To elucidate normal biological variation, we compared ion concentrations in our biobanked maternal serum samples to ion concentrations measured in quality controls serum samples before being freezer stored. These baseline serum samples were provided from 24,199 non-pregnant women from a separate cohort collected between 2015 to 2019. The baseline samples were centrifuged at 21°C, 3100 RPM, 10 min and stored for <24 hours in a climate chamber prior to measuring the ions. As provided by the laboratory, the normal biological ranges in serum for healthy adults are Na^+^_non-pregnant adults_: 136–145 mmol/L and Cl^-^_non-pregnant adults_: 98–107 mmol/L [19].

Measurements from both biobanked serum and baseline quality control serum were performed at the same laboratory and using same methods and analytical instrument, although in different years.

### Statistical analyses

Mean and median concentrations of ions (overall and yearly) with respective standard deviations and 25^th^-75^th^ percentiles were calculated and compared directly to the respective normal biological ranges of each ion in second trimester of pregnancy. Ion concentrations according to storage time were visualized (overall and yearly) and coefficients of determination (R^2^) were calculated for trends over time (27–37 years of storage). For comparison, ion concentrations of baseline quality control samples were also visualized. All analyses were performed in Excel (Microsoft Office 15) and SAS Studio (2018, SAS Institute Inc., Cary, NC, USA).

## Results

In the set of 275 randomly selected biobanked serum samples, the minimum to maximum freezer storage time was 27 to 37 years. The overall mean ion concentrations for the entire storage period were 147.5 mmol/L for Na^+^ and 109.7 mmol/L for Cl^-^ (Table 1). A large proportion of individual ion concentrations were measured outside the normal biological ranges in second trimester for the respective ion of interest: 38.9% for Na^+^; and 43.6% for Cl^-^. Of these, the majority (Na^+^/Cl^-^: 106/116) were *above* normal ranges while only one for Na^+^ and four for Cl^-^ were below. The proportion of ion concentrations outside normal biological ranges were highest in sampling year 1990, corresponding to 32 years of storage, for both Na^+^ (80%) and Cl^-^ (100%). According to concentrations for specific sampling years, a noteworthy high *within year* variation compared with normal ranges was observed across all sampling years, for both ions (Table 1, S1A and S1B Fig).

**Table 1:** Serum concentrations of Na^+^ and Cl^-^ in 275 biobanked pregnancy serum samples according to sampling year and compared to 24,199 baseline serum samples from non-pregnant women.

			Na^+^			Cl^-^		
	Storage time (years)	Serum *n*	Outside normal range^a^ *n* (%)	Mean (SD)	Median (p25-75)	Outside normal range^b^ *n* (%)	Mean (SD)	Median (p25-75)
*Biobanked pregnancy serum samples with storage*								
**Overall**		275	107 (38.9)*	147.5 (10.5)	146.0 (142.0–152.0)	120 (43.6)*	109.7 (8.1)	107.9 (105.2–112.7)
**Yearly**								
1985	37	25	12 (48.0)	146.5 (7.6)	148.0 (139.0–153.0)	12 (48.0)	109.5 (4.6)	108.5 (105.9–113.5)
1986	36	25	14 (56.0)	149.1 (10.0)	149.0 (144.0–158.0)	9 (36.0)	106.7 (5.6)	107.6 (102.2–111.0)
1987	35	25	2 (8.0)	137.3 (6.1)	137.0 (134.0–140.0)	5 (20.0)	103.3 (4.4)	101.8 (101.0–105.6)
1988	34	25	1 (4.0)	140.2 (4.3)	141.0 (137.0–143.0)	2 (8.0)	105.9 (2.6)	106.6 (103.9–107.3)
1989	33	25	7 (28.0)	147 (6.0)	145.0 (143.0–149.0)	5 (20.0)	105.4 (6.1)	104.4 (101.6–106.9)
1990	32	25	20 (80.0)	155.4 (8.5)	153.0 (150.0–159.0)	25 (100)	117.6 (7.3)	115.5 (112.3–120.8)
1991	31	25	14 (56.0)	151.9 (10.4)	150.0 (144.0–157.0)	15 (60.0)	114.5 (8.8)	113.3 (107.3–119.1)
1992	30	25	10 (40.0)	151.8 (17.8)	147.0 (142.0–154.0)	12 (48.0)	113.7 (11.9)	108.7 (107.2–114.7)
1993	29	25	11 (44.0)	149.4 (13.1)	148.0 (143.0–151.0)	13 (52.0)	112.0 (11.1)	109.1 106.8–112.1)
1994	28	25	10 (40.0)	147.5 (4.5)	147.0 (145.0–150.0)	15 (60.0)	110.5 (4.9)	109.6 (107.3–112.7)
1995	27	25	6 (24.0)	145.9 (6.1)	145.0 (143.0–147.0)	7 (28.0)	107.4 (4.0)	106.8 (104.9–116.5)
*Baseline female serum samples without storage*								
2015–2019	0	24199	906 (3.7)^#^	139.4 (2.2)	140.0 (138.0–141.0)	1049 (4.3)^#^	102.1 (2.2)	102.1 (100.7–103.4)

^a^Normal ranges for sodium, Na^+^ in second trimester of pregnancy (129–148 mmol/L) and in non-pregnant adults (136–145 mmol/L).

^b^Normal ranges for chloride, Cl^-^ in second trimester of pregnancy (97–109 mmol/L) and in non-pregnant adults (98–107 mmol/L).

*Na^+^ in biobanked serum: One sample (0.9%, from 1987) was *below* the lower limit of the respective normal ranges, while the remaining 106 samples (99.9%) were above normal ranges.

*Cl^-^ in biobanked serum: Four samples (3.3%, from 1986, 1987, 1987, 1989) were *below* the respective normal ranges, while 116 (96.7%) were above normal ranges.

^#^Na^+^ in baseline serum: 827 (91.2%) of the total 906 samples outside normal ranges were *below* the lower limit of the respective normal ranges.

^#^Cl^-^ in baseline serum: 751 (71.6%) of the total 1049 samples outside normal ranges were *below* the lower limit respective normal ranges.

**Fig 1:**
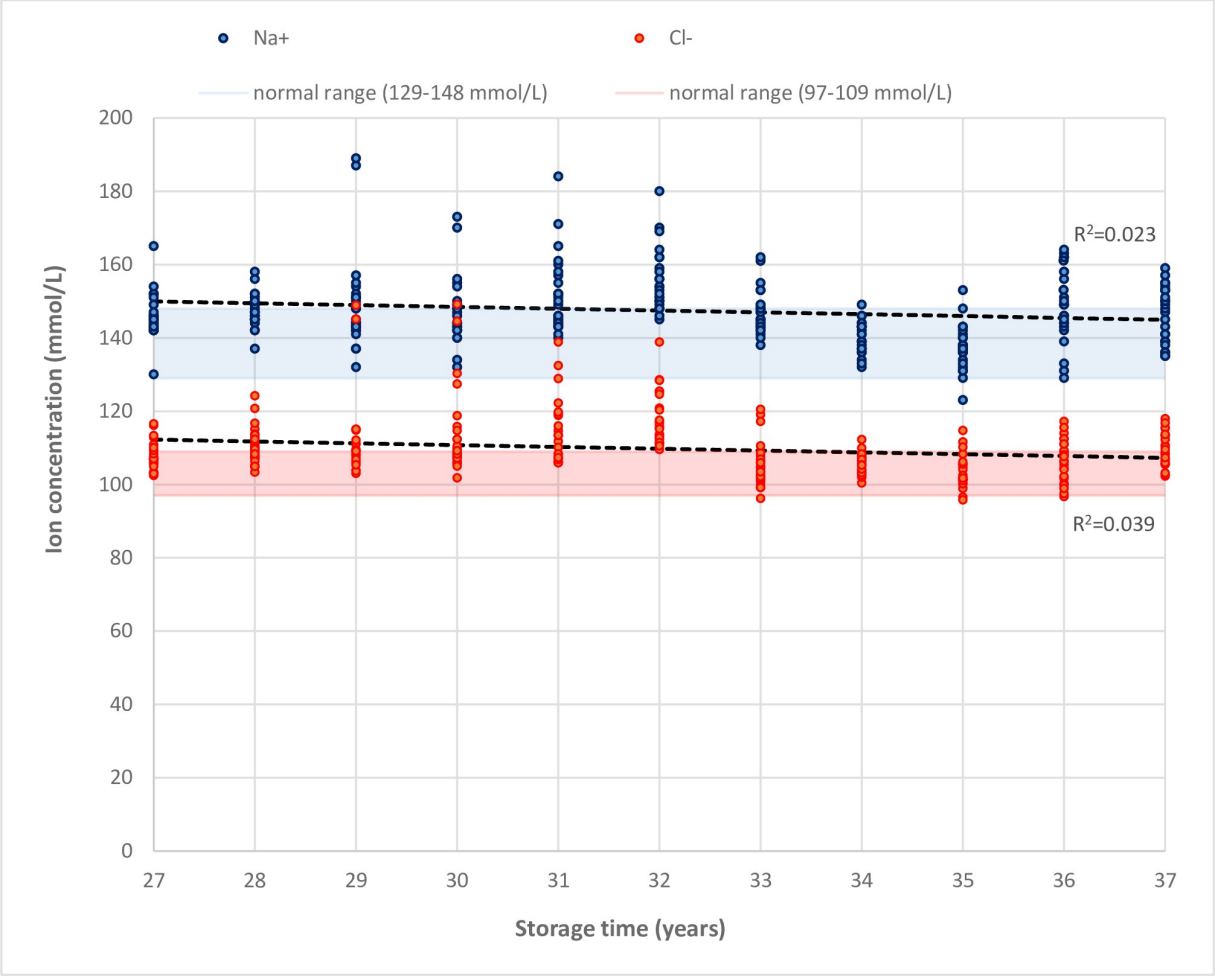
The concentrations of sodium, Na^+^ and chloride, Cl^-^ (mmol/L) in biobanked serum samples from pregnant women (*n* = 275) according to storage time in years and compared to normal biological ranges in second trimester of pregnancy.

There were no marked secular (linear) trends in the concentrations according to storage time for any of the investigated ions, with R^2^ coefficients of 0.023 for Na^+^ and 0.039 for Cl^-^ (Fig 1). Some single sampling years appeared to have more individual samples with very high concentrations (outliers), both evident for Na^+^ and Cl^-^, for which reason trends in concentrations of samples were not entirely stable across years. We *post hoc* checked some of these individual outliers to confirm their visual conditions; some outlier samples had sufficient volume, while others were observed to have a very low volume, which may have led to a relatively higher evaporation, thus potentially resulting in increased concentrations. However, we observed no consistent differences related to sample volume.

**Fig 2:**
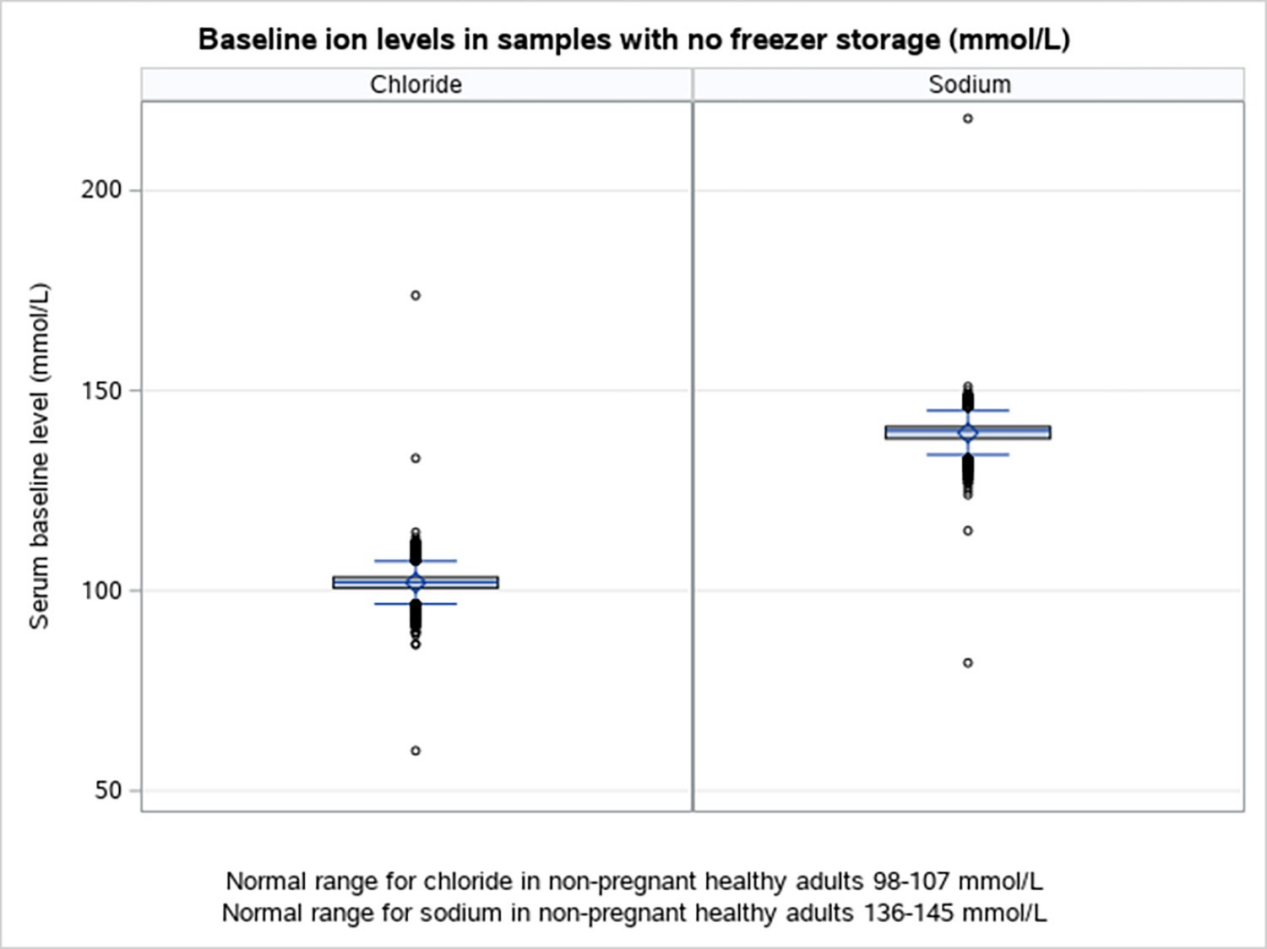
The concentrations of sodium, Na^+^ and chloride, Cl^-^ (mmol/L) in baseline serum samples before freezer storage.

Considering the baseline serum samples in non-pregnant women, a much lower proportion was outside the normal biological ranges for non-pregnant healthy adults. Specifically, 906 (3.7%) and 1049 (4.3%) were outside normal ranges for Na^+^ and Cl^-^, respectively (Table 1). In contrast to biobanked serum samples, these baseline serum concentrations were mainly *below* the respective normal biological ranges (Na^+^/Cl^-^: 91%/72%). Some extreme variation in the individual concentrations was also observed (Fig 2).

## Discussion and conclusions

In this study investigating evaporation of biobanked maternal serum stored at -20°C between 27 to 37 years, we observed no marked linear effects on ion concentrations of Na^+^ and Cl^-^ according to freezer storage time. However, we did observe elevated concentrations of both ions compared to respective normal biological ranges in second trimester of pregnancy, implying some degree of evaporation of the samples. Given the sample handling procedure at the time without immediate freezing, the evaporation may very well have happened even before biobank storage. But without having samples stored shorter periods of time as a comparison, such as within the 0-to-27-year period, it remains unknown if very long-term storage impacts evaporation or pre-analytical factors play more of a role. Although independent of storage time, the higher concentrations of ions were inconsistent for some sampling years and the overall variation in concentrations in biobanked serum were somewhat larger than the normal biological variation observed in baseline serum prior to storage.

Na^+^ and Cl^-^ are regulated within a relatively narrow reference range in healthy individuals and abnormal levels therefore serve as a good proxy for evaporation of serum after years of freezing. Thus, the pattern of results with noteworthy high ion concentrations, especially for specific sampling years, may suggests evaporation and indicate that samples were not identically affected by storage and/or pre-analytical factors. We recognize these concerning discrepancies, but given the small sample size, we were not able to expect ion concentrations to cluster around the mean normal range value. It is not possible to determine if any conditions changed in the sample handling procedure around year 1990 to explain the slightly higher concentrations but it may also be a coincidence with only few samples investigated per year. The relatively large *within year* variation observed across all sampling years is suspected to be caused by the low volume of some samples, but differential serum volume does not explain the entire variation. The baseline serum ion concentrations measured prior to storage used as quality controls also confirm that some normal variation may be expected, although the two data sets are inherently difficult to compare, as the baseline data consider non-pregnant women, while pregnant women are known to have higher variability in Na^+^ and Cl^-^ serum concentrations (wider normal ranges). We acknowledge the high concentrations and large variations observed *within* and *between* years but the reasons for these are largely speculative and may have been caused by a number of pre-analytical factors, where storage time is simply be one of them.

The effect of certain storage and sample handling conditions on the stability of frozen biospecimen has already been studied in various contexts [3,15,20–25], typically to validate bioresources prior to analytical processes. A systematic review from 2014 [3] evaluated current literature relevant to the stability of specific biomarkers in human fluid and determined potential variations associated with different storage temperatures (-20 to -196°C). Another study [26] has postulated that pre-freezing handling such as time and transport until processing/freezing may be factors contributing to variability in concentrations. Effects of different procedures in the laboratory prior to chemical analyses can also lead to erroneous results and represent a pre-analytical bias, including insufficient mixing of thawed serum, insufficient mixing during previous pipetting events, pipetting manually or with the use of robots, accidentally loose lids, variability in tubes, volume and gel etc. [27–29]. Taken together, all these observations from previous studies demonstrate the importance of pre-analytical activities on the usefulness of biobanked samples for epidemiological research. In the present study, we were unable to investigate these specific sample handling protocols and storage conditions, but we acknowledge that the pre-analytical phase should be considered in order determine the reliability of any results of serum sample analyses. Evaporation is simply one factor that can contribute to analytical error. Noteworthy, our biobanked samples were handled by the same protocol, and any variability is expected to be the same across cases and controls, reducing any systematic exposure misclassification bias in future disease-oriented epidemiological studies.

A limitation of our study was that investigating repeated measurements of ion concentrations or comparing with ion concentrations in fresh serum from the same individuals was not possible. This would have been preferred in order to accurately quantify the degree of evaporation. In addition, we were not able to measure ion concentrations in the entire pregnancy screening registry due to costs and prioritization in the use of valuable biobanked biospecimen. We acknowledge that it would be preferable to measure individual ion concentrations for all samples and thereafter adjust each sample based on expected evaporation rate accordingly. But we do not expect this to be a substantial problem given the large number of available samples within the cohort of biobanked samples and since our future analyses will aim at investigating general trends at the group level and not biomonitoring trends according to the individual exposures. Another limitation is that the shortest storage time was 27 years so we could not explore the full range from before storage up to 37 years of storage. This may have provided more insight into effects of long-term storage versus short-term storage of several years.

In conclusion, the elevated ion concentrations of biobanked maternal serum samples stored at -20°C were independent of storage duration in the present study (27 to 37 years). On this basis, we do not recommend applying a duration-depended correction factor to quantified chemical concentrations in future epidemiological studies using these samples. The generally high ion concentrations often outside normal biological reference ranges in second trimester may suggest some evaporation of the samples, especially for specific sampling years, but we speculate that other unknown pre-analytical factors also influence the variations in concentrations. Insufficient serum volume may be of particular concern in this context. On balance, we consider the biobanked serum samples internally comparable over the investigated period of biobank storage and of sufficient quality to be used in future epidemiological studies. Close matching for sampling year can be a necessary approach in the research design. Further, optimal pre-analytical procedures such as sufficient volume and mixing prior to chemical analysis are important components for obtaining reliable chemical results.

## Supporting information

S1 Fig **a.** The concentrations of sodium, Na^+^ in biobanked serum samples from pregnant women according to specific sample years. **b.** The concentrations of chloride, Cl^-^ in biobanked serum samples from pregnant women according to specific sample years.
(TIF)
