# Supplementary figures and images for: Evaporation of serum after long-term biobank storage: A chemical analysis of maternal serum from a large Danish pregnancy screening registry
Source: PLoS One. 2023 Oct 26;18(10):e0293527. doi: 10.1371/journal.pone.0293527 (PMC10602309; doi:10.1371/journal.pone.0293527)

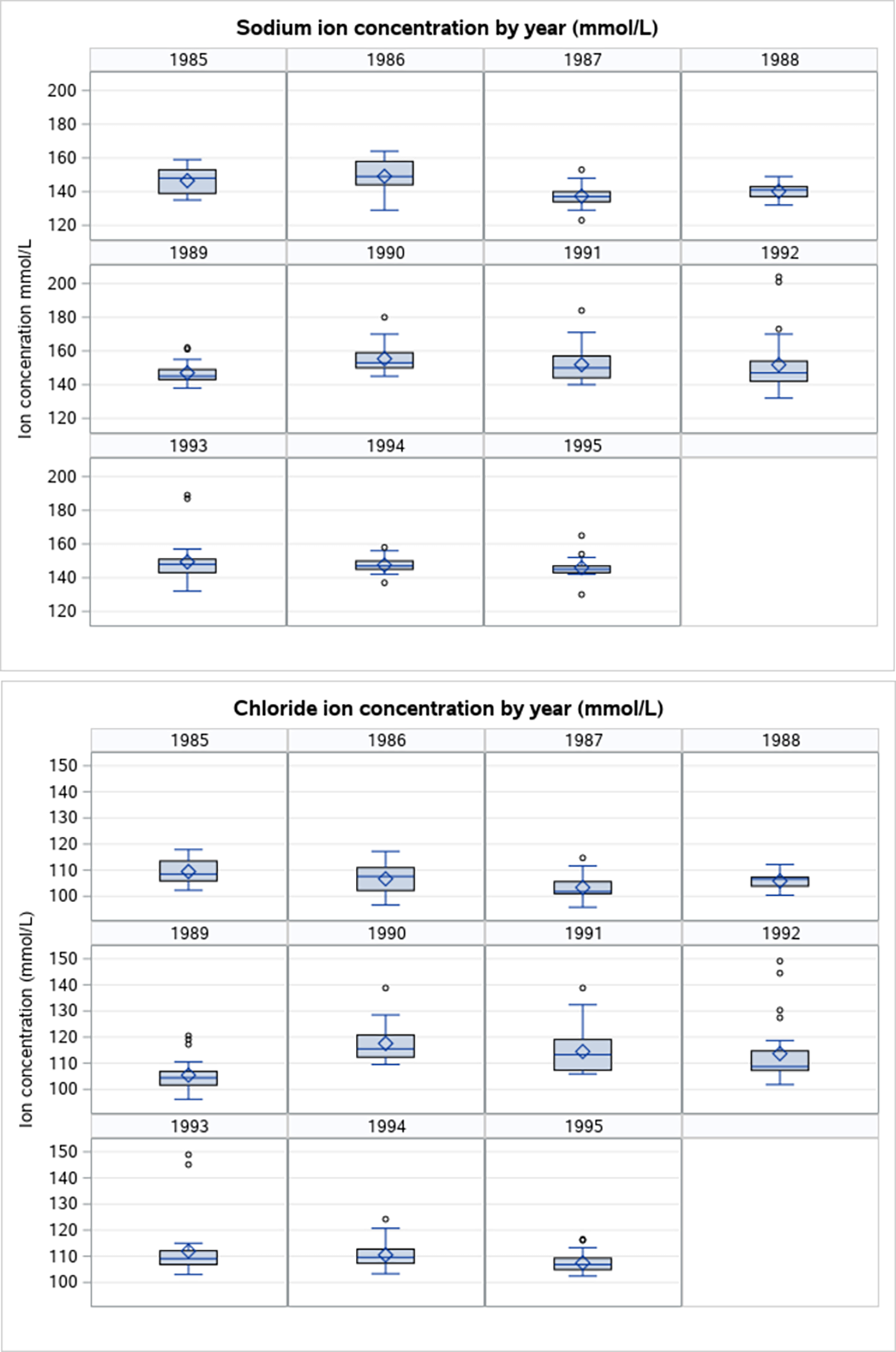

Supplement: S1 Fig — a. The concentrations of sodium, Na+ in biobanked serum samples from pregnant women according to specific sample years. b. The concentrations of chloride, Cl- in biobanked serum samples from pregnant women according to specific sample years. (TIF) [file pone.0293527.s001.tif]
